# Fast and accurate quantification of double-strand breaks in microsatellites by digital PCR

**DOI:** 10.1093/biomethods/bpaf059

**Published:** 2025-08-09

**Authors:** Cécile Palao, Adèle Kovacs, Maria Teresa Teixeira, Guy-Franck Richard

**Affiliations:** Département Génomes & Génétique, Institut Pasteur, Université de Paris, CNRS UMR 3525, 25 rue du Dr Roux, 75015, Paris, France; Sorbonne Université, Collège Doctoral, 75005, Paris, France; Laboratoire de Biologie Moléculaire et Cellulaire des Eucaryotes, Institut de Biologie Physico-Chimie, Sorbonne Université, CNRS UMR 8226, 75005, Paris, France; Département Génomes & Génétique, Institut Pasteur, Université de Paris, CNRS UMR 3525, 25 rue du Dr Roux, 75015, Paris, France; Laboratoire de Biologie Moléculaire et Cellulaire des Eucaryotes, Institut de Biologie Physico-Chimie, Sorbonne Université, CNRS UMR 8226, 75005, Paris, France; Département Génomes & Génétique, Institut Pasteur, Université de Paris, CNRS UMR 3525, 25 rue du Dr Roux, 75015, Paris, France; NARYA Genome Consulting, 93 avenue de Choisy, 75013, Paris, France

**Keywords:** DNA double-strand breaks, fragile sites, non-B DNA structures, repeat expansion disorders, digital PCR

## Abstract

DNA double-strand breaks (DSBs) represent critical events in genome integrity, arising from both endogenous cellular processes and exogenous factors. These breaks are implicated in various genomic aberrations and chromosomal rearrangements, leading to cancers and genetic disorders. Common and rare fragile sites, containing repetitive elements and non-B DNA structures, are particularly prone to breakage under replication stress, which play a pivotal role in cancer development and genetic diseases.

Accurate quantification of DNA breaks in the context of repetitive sequences such as microsatellites or non-B DNA structures is technically challenging. We have been comparing four different methods to reliably quantify DSBs in repetitive DNA, namely Southern blot, DSB-PCR, real-time DSB-qPCR, and digital PCR (dPCR). We show here that dPCR offers enhanced sensitivity and specificity compared to other methods. This provides significant applications for future disease diagnosis, understanding molecular mechanisms generating chromosomal breakage and for the development of gene therapies for microsatellite expansion disorders.

## Introduction

DNA double-strand breaks (DSBs) can occur in the genome for multiple reasons. Endogenous or exogenous factors are responsible for this detrimental type of DNA damage. Among exogenous factors, ionizing radiations, commonly used in chemotherapy, are known to cause DSBs. Metabolic processes such as DNA replication are also a potential cause of DSBs, particularly when replication forks encounter obstacles or collapse, leading to a transient inability to complete this metabolic process. Non-B DNA structures, often at repetitive sequences, are prone to replication fork stalling [1–3]. Additionally, conflicts between replication and transcription can arise, especially when R-loops form, creating a three-stranded structure that can interfere with replication and potentially induce DSBs. The three-dimensional structure of the genome can also contribute to DSB formation, particularly through chromatin loop anchor points [4]. They serve as sites of interaction between distant regions of the genome and can be hotspots for DSBs. Importantly, these anchoring sites are often enriched in specific DNA structures called G-quadruplexes (G4), which can predispose to DSB formation due to their ability to interfere with normal cellular processes [5]. An unrepaired DSB can be detrimental to genome integrity, potentially leading to errors, gross chromosomal rearrangements, loss of heterozygosity, or telomere-to-telomere fusion if telomeres become deprotected. These events are hallmarks of cancers or may lead to the creation of oncogenic genes through chromosomal translocations.

In human cells, common fragile sites (CFSs) and rare fragile sites (RFSs) break under replication stress and are potential hotspots for chromosomal rearrangement. Some of the CFSs typically contain AT-rich sequences, whereas RFSs rather contain expanded CGG repeats, both can form hairpin-like secondary structures [6]. They may cause replication fork stalling and chromosomal breakage, as observed in humans and budding yeast [7]. Breakage at CFSs can lead to in vivo amplification of human oncogenes [8] and deletion of tumor suppressor genes [9], like *FRA3B*, the most studied CFS. This evidence shows the implication of CFSs in cancers, as well as in disorders such as Fanconi Anemia and Bloom Syndrome. FANCM is a translocase that enables CFSs stability but is mutated in Fanconi Anemia. FANCM uses its translocase activity to promote fork reversal to remove secondary structure of AT-rich CFSs when ssDNA is formed during replication, therefore preventing DSB formation [10]. BLM, which is mutated in Bloom Syndrome, is a helicase proposed to resolve DNA secondary structures of AT-rich CFSs but differently from FANCM and works together with FANCM to preserve the integrity of CFSs [11]. RFSs are less studied than CFSs, they contain CGG trinucleotide repeat expansions in the Fragile X Syndrome, the Jacobsen Syndrome, and other mental retardation syndromes [12]. The integrity of fragile sites is therefore very important to allow genome maintenance, and molecular tools to accurately quantify DNA breaks at fragile sites are currently lacking.

Among the techniques used, the comet assay allows for the quantification of DSBs in individual cells but does not directly distinguish between DSBs and single-strand breaks (SSBs) [13]. Additionally, the comet assay does not provide information on the specific location of DSBs within the genome. To achieve this, it must be coupled with FISH [14]. Southern blot, another classical method for quantifying DNA breaks, is time-consuming, is semi-quantitative depending on the detection method used, but allows to identify all recombination intermediates [15]. END-seq is another technique allowing to detect every DSB in the genome, their genomic location and to quantify them [16]. In addition to END-seq, several other NGS-based methods such as BLESS, i-BLESS, BLISS, and INDUCE-seq have also been developed to map DSBs genome-wide with high resolution [17–20]. However, the sequencing may be challenging within a repeated sequence and could bias the quantification. Therefore, a quantitative and easy-to-run method to detect DSBs, especially in repetitive DNA, is needed.

Our lab has been working for many years on microsatellite expansion disorders. Some of these sequences, such as CTG trinucleotide repeats, can form stable secondary structures in vitro [21]. Expansion of CTG triplet repeats in the 3ʹ UTR of the *DMPK* human gene leads to a disease called myotonic dystrophy type 1 (DM1 or Steinert disease). This neuromuscular disorder is inheritable and prone to expansion in offsprings [22]. We have been using DSBs induced by a TALE nuclease (TALEN) in an expanded CTG tract, to shorten the expanded repeat tract, as a possible approach to gene therapy [23–25].

**Figure 1.**
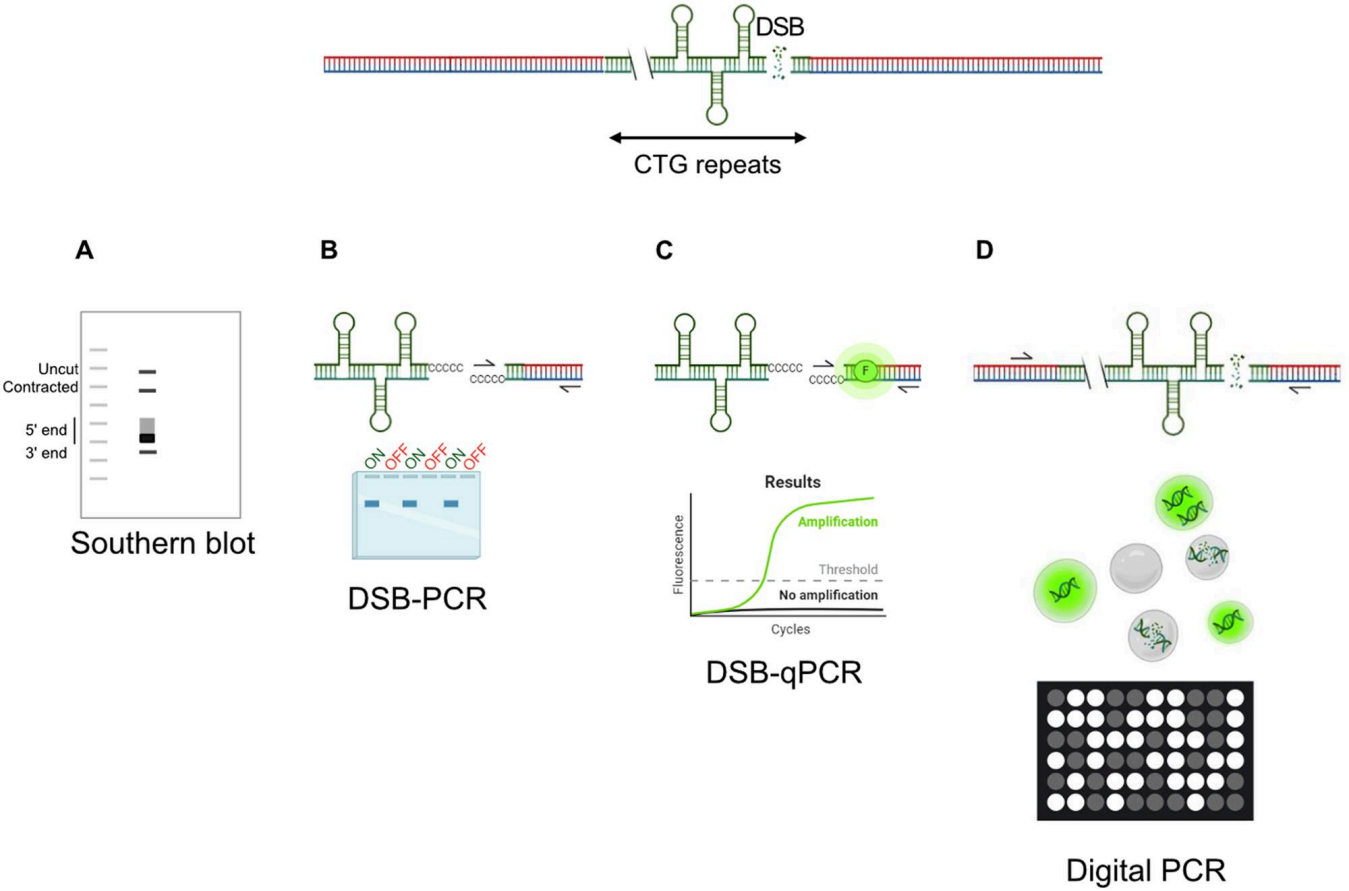
Overview of four different methods used to quantify a DSB within a CTG trinucleotide repeat. **A**: Southern blot using specific probes reveals different DNA products, the uncut allele, the contracted allele, the 5′ and 3′ ends of the DSB. **B**: Detection of DSBs using DSB-PCR. DSB-PCR allows qualitative detection of the DSB through PCR amplification after terminal transferase-mediated polyC tailing. A product is only observed when the DSB is present (ON). **C**: DSB-qPCR enables DSB quantification by real-time PCR using the same primers as in panel B, with SYBR Green fluorescence and comparison to a standard curve. **D**: Digital PCR (dPCR) provides absolute quantification by partitioning DNA into droplets. Amplification of unbroken CTG repeats (EvaGreen, blue channel) and a control gene (*JEM1*, HEX probe, green channel) allows calculation of the percentage of broken molecules.

In the present work, we have used different molecular methods to accurately quantify DNA breaks in a long CTG trinucleotide repeat. Southern blot, DSB-PCR, real-time DSB-qPCR, and digital PCR were compared. An overview of these four approaches is provided in Fig. 1A–D. The DSB-PCR method is based on the Telo-PCR protocol that was first described by the Lingner’s lab and used to determine the length of a unique telomere by analyzing the size of a PCR product [26]. We previously adapted this method to amplify a DSB at a specific locus [25], which we then termed DSB-PCR. Telo-PCR has been subsequently refined to measure telomere length by qPCR, by comparing the Ct value of a telomere to a single copy gene [27], or to a standard curve made with different concentrations of a standard oligomer of a single-copy gene [28]. Here, we adapted the DSB-PCR method to amplify a DSB at a specific locus using qPCR and compared the result to a standard curve to determine the percentage of DSB. This adapted method was subsequently named DSB-qPCR. We finally assayed a novel method using digital PCR for absolute quantification, particularly in the context of repeated sequences that can form secondary structures that are known to be hard to amplify. We show here that dPCR is the most effective method to quantify a DSB in a CTG trinucleotide repeat, by allowing perfect and quantitative amplification through the microsatellite. It is easy, fast, and cheap compared to Southern blot and can even detect a very small percentage of the target, reaching a precision that no other available method can attain.

## Materials and methods

### Yeast strain

The *Saccharomyces cerevisiae* strain used in the experiments was the strain 6162-3D containing 80 CTG repeats inserted in *SUP4* gene as previously described [29]. The DSBs in the CTG repeats were made by inducing TALEN expression. The 6162-3D strain was transformed with two plasmids, pCLS9996 and pCLS16715, respectively, coding the right and left arm of the TALEN and have also been previously described [24]. Expression of both TALEN arms was regulated by a TetOFF promoter and occurred in the absence of doxycycline in the culture medium.

### DSB analysis by Southern blot

Cells were collected 24 hours after TALEN induction. This time point was selected based on its routine use in our laboratory for previous experiments involving TALEN-induced breaks at CTG repeats, where it reliably allows for the detection of DSBs. Total genomic DNA (4 μg) from TALEN-induced cells was digested for 6 hours by *Eco*RV (20 U) (NEB), purified using AMPure XP beads (Beckman Coulter) and then migrated on a 1% agarose gel for 16–17 hours at 1.5 V/cm. The gel was transferred on a nylon charged membrane by vacuum blotting, after depurination using HCl (0.25 M) (30 min) and NaOH (0.4 M) for 2 hours to allow the transfer. The membrane was washed with sterile water and then pre-incubated for 1 hour at 65°C under rotation with 10 mL of Church hybridization buffer (250 mM Phosphate buffer pH 7.4, 7% SDS, 1 mM EDTA, 1% BSA). Two probes that hybridize upstream and downstream the CTG repeats were synthesized by PCR using DIG-labeled-dUTP (Roche). su37/su38 primers were used to synthetize the upstream probe and su39/su40 primers to synthetize the downstream probe (Supplemental Table S1). The PCR products were then gel purified using the Monarch DNA Gel Extraction Kit and 250 ng of each probe was used for hybridization. Probes were heat denatured at 95°C during 5 min, rapidly put on ice and added to the pre-hybridizing membrane and hybridized overnight at 65°C under rotation. The membrane was then washed twice for 10 min under agitation at 65°C with a washing buffer (20 mM Phosphate buffer pH 7.4, 1% SDS, 1 mM EDTA). The membrane was subsequently blocked with 100 mL 1× blocking buffer (100 mM Maleic acid, 150 mM NaCl, pH 7.5) and with 5% milk for 1 hour under gentle agitation. Hybridization with the antibody was performed with 10 ml of antibody anti-DIG (Anti-Digoxigenin-AP Fab fragments—Roche) in 100 mL Blocking Buffer and incubated for 1 hour at room temperature under gentle agitation. The membrane was then washed twice with blocking buffer 1× for 15 min and then washed 5 min with Detection Buffer (100 mM Tris, 100 mM NaCl, pH 9.5). It was then incubated 10 min in the dark with CSPD (Roche) and revealed with a ChemiDoc Imaging System from Bio-Rad and using the Image Lab software. Signals were quantified with the same software, using Volume Tools. This protocol is extensively described in a previous study [30].

### DSB qualitative detection by DSB-PCR

A terminal transferase-mediated PCR assay [26] was used to amplify the TALEN-induced DSB; 100 ng genomic DNA was heat-denatured 5 min at 94°C and treated with 7 U of terminal deoxynucleotidyl transferase (Takara) in a volume of 10 μL (100 mM 4-(2-hydroxyethyl)-1-piperazineethanesulfonic acid [HEPES], pH 7.2; 40 mM MgCl2; 0.5 mM DTT; 0.1% BSA; and 1 mM deoxycytidine triphosphate [dCTP]) for 30 min at 37°C to add polyC tails to 3′ OH free ends. The enzyme was inactivated for 10 min at 65°C and 5 min at 94°C. Then, 30 μL of this PCR mix was added to each reaction to obtain a final volume of 40 μL containing 67 mM Tris HCl (pH 8.8), 16 mM (NH_4_)2SO_4_, 5% glycerol, 0.01% Tween, 200 μM each deoxynucleotide triphosphate (dNTP), 625 nM of each primer (G18 and VMS14, Supplemental Table S1) and 2.5 U of DreamTaq (Thermo Scientific). The following PCR program was used: 94°C for 2 min, (94°C for 20 s, 62°C for 12 s, and 72°C for 20 s) for 45 cycles and then 72°C for 5 min. To analyze reaction products, 20 μL was loaded on a 2% analytical agarose gel.

### DSB quantification by DSB-qPCR

DSB-qPCR relies on the same protocol as DSB-PCR. After the Terminal Deoxynucleotidyl Transferase reaction, the DNA was then amplified using the same buffer and primers as DSB-PCR. However, SYBR Green I (ABP-Biosciences) and Rox Reference Dye (ABP-Biosciences) were added to the qPCR reaction to a final concentration of 0.3× and 50 nM, respectively. The following qPCR program was used: 94°C for 2 min (94°C for 20 s, 62°C for 12 s, and 72°C for 30 s) for 55 cycles and then a melting curve was added. The temperature started at 65°C up to 95°C by increasing 0.5°C every 5 s.

To design the standard curve, *Saccharomyces cerevisiae* genomic DNA was digested for 5 hours with 20 U XbaI enzyme (NEB) to create a sample with 100% DSB next to the CTG repeats. A mock reaction was done the same way, omitting the enzyme, to get a 0% DSB undigested control sample. The DNA was purified using AMPure XP beads (Beckman Coulter). The digested and undigested samples were then mixed in different proportions to simulate different percentages of DSB. The final concentration of each tube was 1.5 ng/µL. The different samples were then quantified by DSB-qPCR with a final concentration of 0.15 ng/µL per qPCR reaction.

### DSB quantification by dPCR

All products were purchased from Stilla Technologies, unless specified. dPCR assays were performed using 1 ng genomic DNA per reaction. Reagents from Stilla Technologies were used at the recommended concentrations and volumes. For each dPCR reaction, 1 µL of DNA (1 ng) was used, 2.5 µL Buffer A—naica PCR Mix 10× to a final concentration of 1×, 1 µL Buffer B—100% to a final concentration of 4%, 1 µL Alexa 647 0.02 mg/mL (Invitrogen) at a final concentration of 0.74 µg/mL, 1.9 µL EvaGreen 20× (BioTIUM) at a final concentration of 1.5×, CP19 and upCTGfwd couple of primers at a volume of 0.225 µL each and at a final concentration of 0.9 µM each, CP21 JEM1 and CP22 BIS JEM1f were also used at a volume of 0.225 µL each and at a final concentration of 0.9 µM each. A JEM1 probe was also used at a volume of 0.625 µL per reaction at a final concentration of 250 nM. Primers and the JEM1 probe were ordered from Eurofins Genomics and are described in Supplemental Table S1. All these reagents were in the same reaction. Sterile water was used to complete the reaction to a volume of 25 µL. The mix was loaded in a well of Sapphire chip and the chip was then placed onto a Naica Geode (automated droplet generator and thermocycler). The following dPCR program was used: Droplet Partition at 40°C “Sapphire V1”, 95°C for 15 min, (95°C for 15 s, 55°C for 30 s, 60°C for 35 s) for 45 cycles, Close cycle, and Release P “Sapphire V1.”

The dPCR Sapphire chip was read by a three-color fluorescence reader (Naica Prism3) and the Crystal Reader software was used to adjust the scanning parameters. The results were then analyzed using the Crystal Miner software.

## Results

### Quantification of recombination intermediates by Southern blot

**Figure 2.**
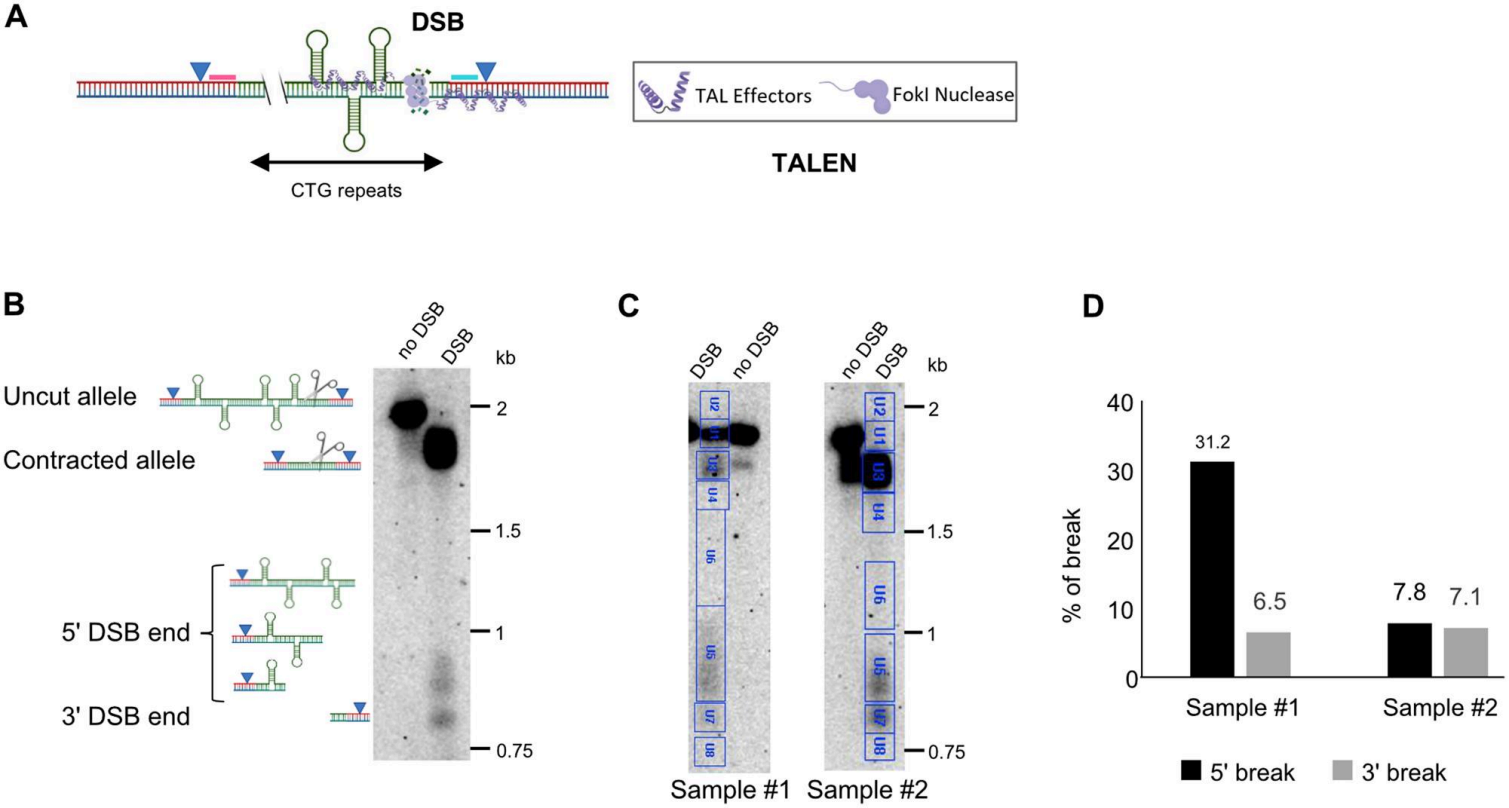
DSB quantification by Southern blot. **A**: Schematic representation of CTG trinucleotide repeats inserted in the genome of *Saccharomyces cerevisiae*. A DSB is induced by a TALEN at the 3ʹ end of the repeat tract. Triangles indicate the locations of *Eco*RV sites used for restriction digestion prior to Southern blotting. Pink and blue rectangles indicate the positions of the 5ʹ and 3ʹ probes, respectively. **B**: Example of Southern blot hybridized with upstream and downstream probes, all recombination intermediates are visible, including the smear on the 5ʹ end of the repeat tract. Triangles indicate the locations of *Eco*RV sites used for restriction digestion prior to Southern blotting. Scissors indicate the DSB location. **C**: Two examples of Southern blots with different percentages of DSBs (see Table 1 for calculations). The signal intensity of the bands and backgrounds within each blue rectangle was measured using the volume tool from Image Lab software, and the data are presented in Table 1. **D**: Percentage of breaks in the CTG repeats for the 5ʹ and the 3ʹ ends, for each DNA sample analyzed. Quantification was performed using the Image Lab software by measuring signal intensities from defined areas (U5 for the 5ʹ break, U7 for the 3ʹ break), with subtraction of corresponding background signals (U6 and U8, respectively). The resulting values were normalized to the total signal intensity of all bands (uncut, contracted, and broken), each with its own background correction. Detailed calculations are shown in Table 1.

Southern blots have been used for decades to quantify a DNA break at a known location in the genome. After DSB induction, DNA is extracted and digested with a restriction enzyme. The samples are then migrated on an agarose gel and transferred onto a nylon membrane. The membrane is hybridized with two probes, upstream and downstream of the DSB. Using two probes allows the detection of both DSB ends: the 5ʹ end is detected by the upstream probe and the 3ʹ end is detected by the downstream probe. In our experimental system, the DSB is induced by a TALEN at the 3ʹ end of a CTG trinucleotide repeat tract integrated in the budding yeast genome (Fig. 2A). The 3ʹ end of the DSB is visible as a unique band around 800 bp (Fig. 2B). The 5ʹ end of the break, containing 80 CTG triplets, undergoes contraction over time. This contraction leads to different repeat lengths visible as a smear. The parental uncut allele appears as a unique band around 2 kb. When the repeat tract is fully contracted after DSB repair, it appears as a smaller band around 1.8 kb. Different types of DNA molecules can be present at a given time: uncut, broken unrepaired, or fully repaired and contracted. The signal of each band is quantified using a Bio-Rad ChemiDoc system with the Image Lab software. The results for two Southern blots with different percentages of DSB are illustrated in Fig. 2C and D and Table 1.

**Table 1.** Southern blot quantification using the Image Lab software.^a^

Sample #1			Sample #2		
Label	Type	Volume	Label	Type	Volume
U1	Full length allele	1 595 996	U1	Full length allele	1 719 524
U2	Background full length allele	304 624	U2	Background full length allele	1 030 168
U3	Contracted allele	1 372 968	U3	Contracted allele	14 037 895
U4	Background contracted allele	528 404	U4	Background contracted allele	1 606 612
U5	5ʹ break	2 446 924	U5	5ʹ break	3 526 660
U6	Background 5ʹ break	1 375 188	U6	Background 5ʹ break	2 321 516
U7	3ʹ break	742 804	U7	3ʹ break	1 634 544
U8	Background 3ʹ break	520 004	U8	Background 3ʹ break	544 948
*(U1–U2) + (U3–U4) + (U5–U6) + (U7–U8)*	Total Volume	3 430 472	*(U1–U2) + (U3–U4) + (U5–U6) + (U7–U8)*	Total Volume	15 415 379
*(U5–U6)/Total Volume*	% 5ʹ DSB	31.2 %	*(U5–U6)/Total Volume*	% 5ʹ DSB	7.8%
*(U7–U8)/Total Volume*	% 3ʹ DSB	6.5 %	*(U7–U8)/Total Volume*	% 3ʹ DSB	7.1%

a Southern blots corresponding to this table are shown in Fig. 2.

At the present time, a Southern blot is the only available molecular method allowing to detect all recombination intermediates during DSB-repair [31]. However, when using non-radioactive probes (such as DIG-labeled DNA), the response is not always strictly linear, as was previously shown by our lab [30]. This may lead to important variations between the 5ʹ and the 3ʹ end quantifications, although they should theoretically be identical (Fig. 2C and D).

### DSB-PCR, a method to amplify a single DSB at a specific location

**Figure 3.**
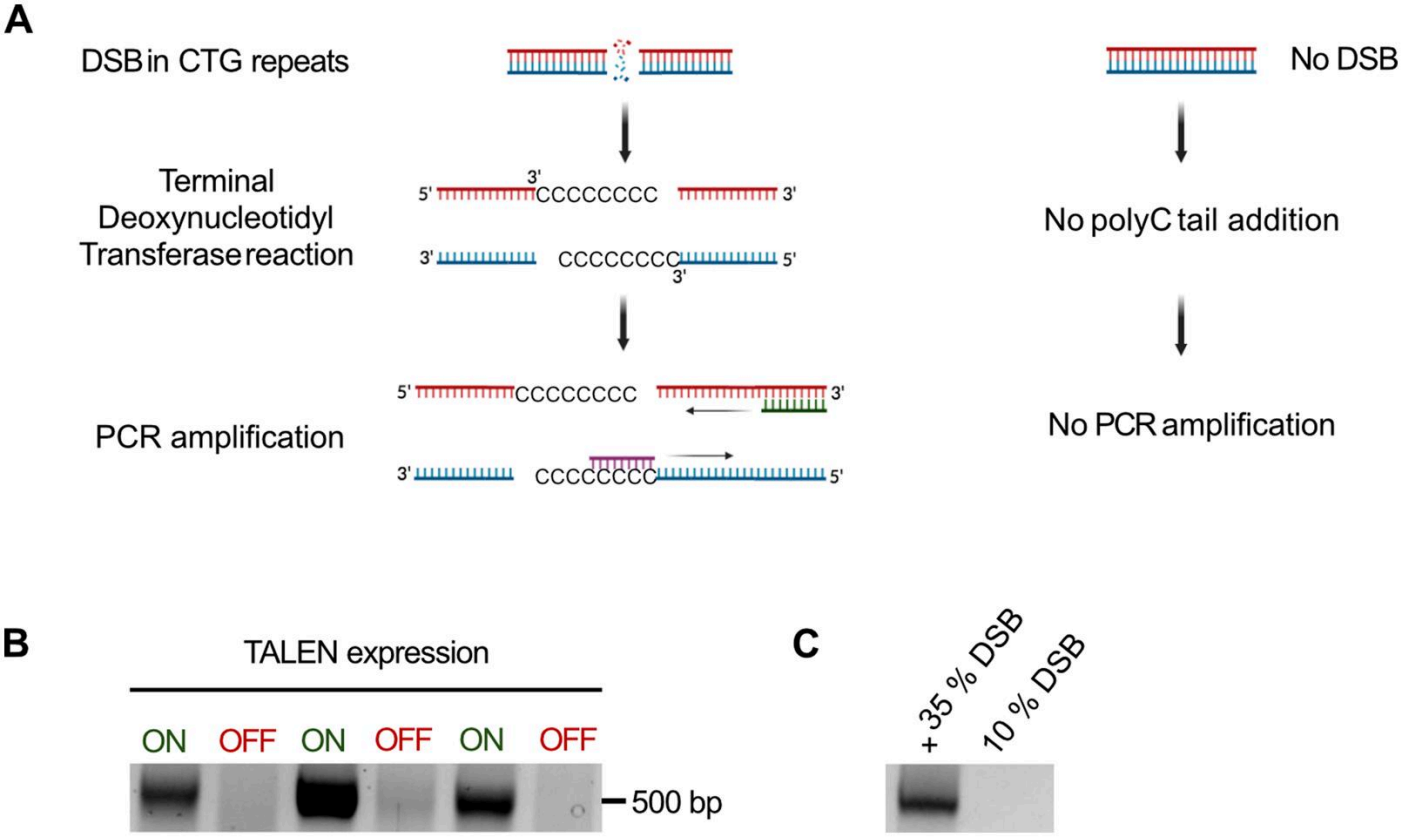
Detecting a DSB by DSB-PCR. **A**: Schematics of DSB-PCR. After DSB induction, the DNA is extracted, and a Terminal Deoxynucleotidyl Transferase reaction (TdT) is performed using dCTP. PCR amplification uses two primers, one recognizing the polyC tail (G18) and a specific sequence downstream of the DSB (VMS14). If no DSB is present in the DNA, no polyC tail is added, and nothing is amplified by PCR. **B**: PCR products are run on a 2% agarose gel. DNA with a DSB induced (ON) by TALEN in CTG repeats is visible with a band around 500 bp. There is no amplification product when the TALEN is not induced (OFF). **C**: The same experiment is run on genomic DNAs with a known percentage of DSB, as determined by Southern blot (35% and 10% DSB, respectively).

Telo-PCR was initially described by the Lingner’s lab and used to measure the length of a single telomere. This method has been adapted by our lab to detect a unique induced DSB in a CTG trinucleotide repeat [25] and then named DSB-PCR. After in vitro addition of a polyC tail to 3ʹ DNA ends using Terminal Deoxynucleotidyl Transferase (TdT) in the presence of dCTP, PCR is performed with a polyG primer recognizing the polyC tail and a specific primer recognizing a sequence upstream of the DSB (Fig. 3A). If a break occurs at this specific locus, a unique band is revealed. Upon induction of a TALEN, a DSB is detected in the CTG repeats as a band around 500 bp. Without TALEN induction, no DSB occurs and no band is visible (Fig. 3B). Although the polyG primer used in this assay contains consecutive guanines, which are theoretically capable of forming G-quadruplex structures that could hinder primer annealing or extension, no such interference was observed under our experimental conditions. This is consistent with our previous results [25], where the same primer design was shown to function efficiently. However, this method has limitations. Small amounts of DSBs at specific genomic locations (10%) may not be readily detectable, rendering the technique qualitative rather than quantitative (Fig. 3C).

### DSB-qPCR, a quantitative variation of DSB-PCR

**Figure 4.**
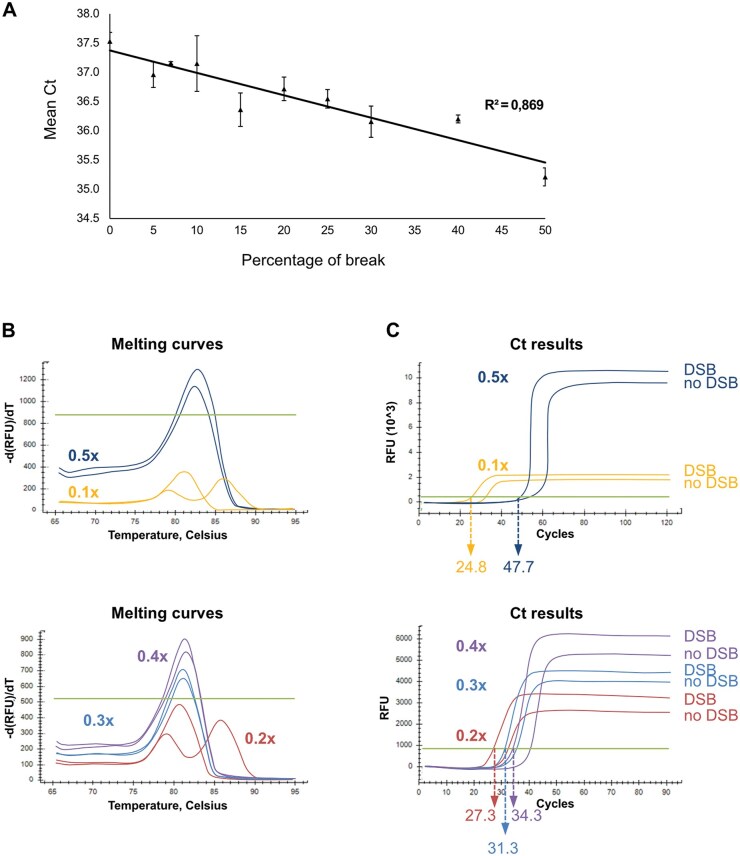
Quantifying a DSB by DSB-qPCR. **A**: Standard range of the mean Cycle threshold (Ct) depending on the percentage of break, as determined by three independent experiments. Genomic DNAs containing different percentages of DSBs were made with the XbaI restriction enzyme. A TdT reaction was performed on this DNA and then amplified by qPCR using the same primers as DSB-PCR (G18 and VMS14). The mean Ct of the triplicate experiments was calculated, and a trendline was plotted on the graph. The correlation coefficient is indicated. **B**: Melting curves of DSB-qPCR performed with different concentrations of SYBR Green I, on genomic DNA with or without a DSB. **C**: Ct values of DSB-qPCR performed with different concentrations of SYBR Green I, in the presence or absence of a DSB. Ct values are shown for DNA containing a DSB.

The search for a new method to quantify small amounts of DSBs led us to adapt real-time qPCR to DSB-PCR. A polyC tail was added to free 3ʹ ends, similarly to DSB-PCR. Using the same primers as in DSB-PCR, a real-time qPCR was performed. The same non-commercial buffer described by the Lingners’ lab was utilized [26], with the addition of SYBR Green and Rox (see Materials and Methods section). Since Ct values do not directly provide a percentage of breaks, a standard curve is required. We have been using different amounts of broken molecules obtained by enzymatic restriction near the CTG trinucleotide repeats. Digested and undigested genomic DNAs were mixed to generate the standard curve shown in Fig. 4A. Subsequently, DSB-qPCR was performed on these samples. To determine the percentage of DSBs within the CTG repeats of a new sample, the exact same protocol was followed, and Ct results obtained from experiments were plotted on the standard curve.

However, the DSB-qPCR method has some limitations. The use of a non-commercial buffer requires testing different concentrations of reagents to optimize conditions. We found that a 1× concentration of SYBR Green was too high and inhibited the reaction. Concentrations of 0.1–0.2× showed two peaks for the melting curve (Fig. 4B) and could not be used. Higher SYBR Green concentrations exhibited only one peak on the melting curve, but Ct values increased with dye concentration (Fig. 4C). Therefore, the concentration of SYBR Green is crucial and can significantly impact results. Another important consideration is that the Ct depends on the DNA concentration of the sample. The standard range was established with a DNA concentration of 0.15 ng/μL in the qPCR reaction. Therefore, the sample must strictly have the same concentration in the qPCR reaction too. Additionally, the Ct values for different percentages of DSBs are not completely linear (correlation coefficient = 0.869). We therefore concluded that DSB-qPCR shows some limitations restricting its wide use for precise DSB quantification.

### dPCR, an accurate and easy method to quantify a DSB

**Figure 5.**
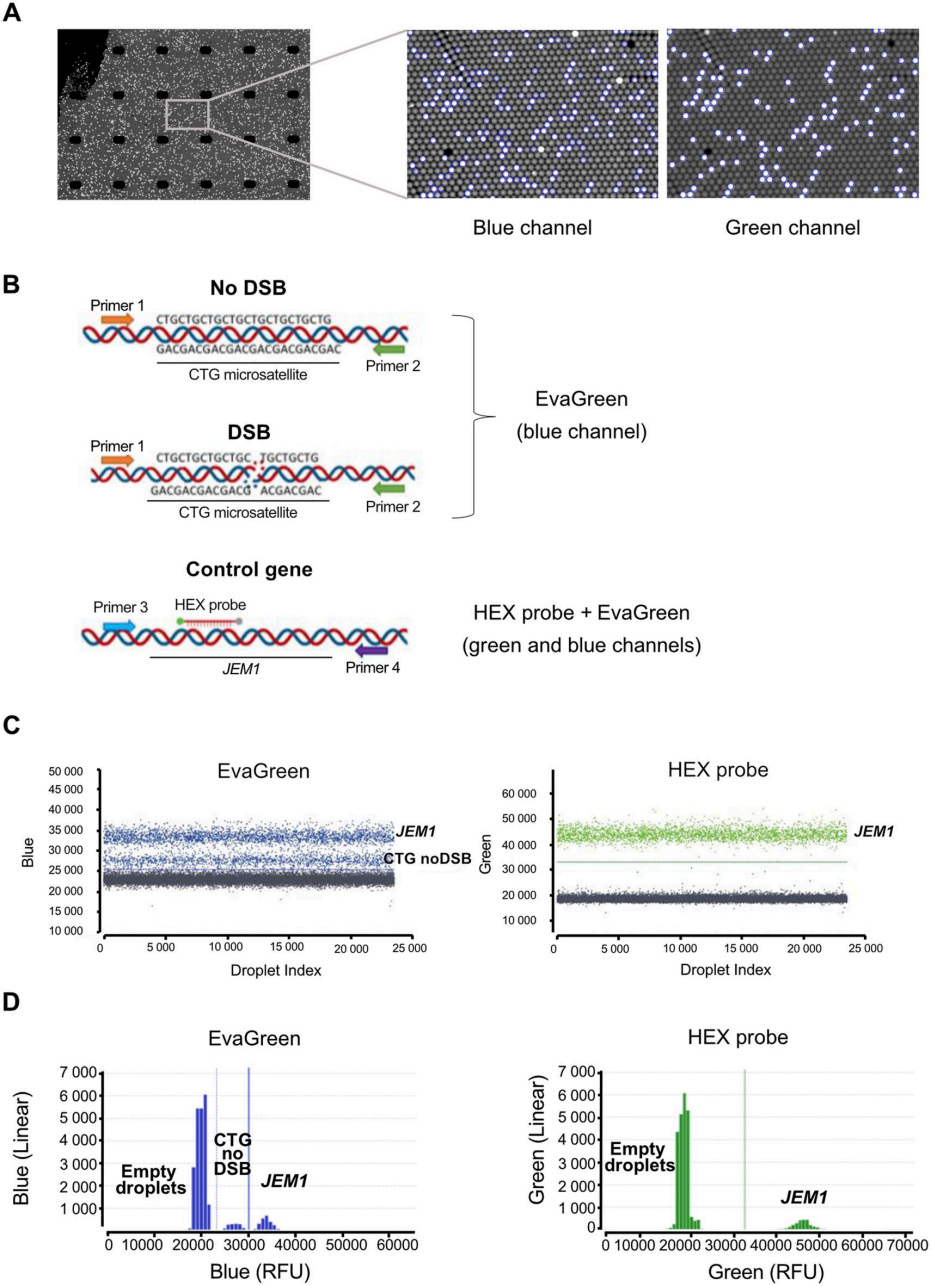
DSB quantification by dPCR. **A**: Image of a dPCR Sapphire chip from Stilla Technologies containing 25 000 droplets in which 0, 1, 2, 3, etc. copies of DNA can be trapped depending on the DNA concentration loaded in the chip. The zoom shows droplets “ON” and “OFF” in the blue and green channel, respectively. “ON” droplets contain one or more DNA copies that have been amplified by PCR resulting in detectable fluorescence in the appropriate channel. “OFF” droplets contain either no DNA or one or more DNA copies that have not been amplified by PCR and resulting in no detectable fluorescence in the appropriate channel. **B**: Schematics of the method principle. In the absence of a DSB, the PCR can amplify the CTG sequence resulting in fluorescence detectable in the blue channel, due to the presence of EvaGreen in the mix. When a DSB is present in the CTG tract, no amplification is possible, and no fluorescence is emitted. A control gene, *JEM1*, is used to determine the number of copies of DNA added in the dPCR chip. The *JEM1* gene is always amplified since it is never broken. A HEX-MG BEQ probe is used to detect *JEM1* gene amplification in the green channel, as a single population. The amplification of this gene can also be detected with the blue channel due to the presence of EvaGreen, resulting in a double population for the blue channel (*JEM1* and CTG repeats with no break). **C**: dPCR graphs for the blue and green channels. The graph for the blue channel represents a double population: amplification of the CTG repeat sequence with no DSB as well as the *JEM1* gene. The threshold can be manually adjusted to separate the two blue populations. See Table 2 for results. The graph for the green channel represents the amplification of the *JEM1* gene only. **D**: Other possible representation of the dPCR results provided by Crystal Miner software. The threshold can also be adjusted in this representation to separate the blue populations.

Digital Polymerase Chain Reaction dPCR is a technique with origins dating back to 1999 [32], but recent technological improvements have made it widely accessible for various applications such as structural variant analysis [33], DSB quantification in the context of a non-repeated sequence [34], or the measurement of viral reservoir represented by HIV DNA and RNA molecules [35]. It is useful in a wide range of molecular applications and is increasingly used due to its high reproducibility and efficacy, without the need for a standard range, as required by qPCR. The dPCR Sapphire chip from Stilla Technologies is composed of 25 000 microdroplets (Fig. 5A). Each droplet can trap a variable number of DNA copies depending on the DNA concentration used in the reaction and following a Poisson distribution. Ideally, only one DNA molecule (or one genome) should be present in each droplet. After the reaction, each droplet is either “ON” or “OFF.” “ON” droplets contain one or more DNA copies that have been amplified by PCR, resulting in detectable fluorescence in the appropriate channel. “OFF” droplets contain either no DNA, or one or more DNA copies that have not been amplified by PCR, resulting in no detectable fluorescence.

To detect a DSB at a CTG repeat tract, we used two primers that recognize sequences upstream and downstream of the repeats. If a break occurs in the CTG repeat tract, no fluorescence will be detected. However, if there is no break, the primers can amplify the CTG sequence, resulting in fluorescence in the blue channel due to EvaGreen (Fig. 5B). The primer pair used to amplify the CTG repeats has been shown by qPCR and dPCR to have a 99% efficacy (Supplemental Figs. S1A-B). A control gene is necessary to determine the total number of DNA copies in the reaction. Therefore, in the same dPCR reaction mix, another couple of primers is used to amplify a control uncut gene (*JEM1*), and a HEX-MG BEQ probe detects *JEM1* and emit fluorescence in the green channel (Fig. 5B). Since the *JEM1* gene can also be detected in the blue channel, the amplification of the unbroken CTG sequence and the *JEM1* locus are represented by two populations in the blue channel (Fig. 5C and D). In the green channel, a single population is seen, representing the *JEM1* amplification. Moreover, we observe that all “ON” droplets in the green channel are also “ON” in the blue channel due to EvaGreen detection of *JEM1*, confirming signal overlap. The additional “ON” droplets visible only in the blue channel correspond specifically to amplification of uncut CTG molecules (Fig. 5A).

Under the Poisson Law, the number of DNA copies/μL that are amplified in each channel can be calculated by the Crystal Miner Software (Table 2). As there are two populations in the blue channel, the number of DNA copies/μL represents the amplification of uncut CTG repeats as well as the *JEM1* amplification. To determine the number of uncut CTG repeats, the number of DNA molecules in the HEX population is subtracted from the number of molecules in the EvaGreen channel (Table 2). From here, the fraction of unbroken CTG repeats can be easily calculated, and then, the percentage of broken molecules can be determined (Table 2).

**Table 2.** Results of the dPCR given by the Crystal Miner software and performed on DNA quantified by Southern blot.^a^

Sample #1							
Population	Nb droplets	**A** Blue channel C(cp/µL)	Nb positive droplets	**B** Green channel C(cp/µL)	Nb positive droplets	**C** % unbroken CTG	**D** % broken CTG
Double population (unbroken CTG + *JEM1*)	23 527	341.8	4516	–	–	Column (A_unbroken CTG_/B_*JEM1*_)×100 = 65.4%	100-Column C = 34.6%
Single population (*JEM1*)	23 527	208.1	2863	206.6	2844		
Single population (unbroken CTG)	23 527	Column (A_unbroken CTG+__*JEM1—*_B_*JEM1*_)= 135.2	1 672	–	–		

Sample #2							
Population	Nb droplets	**A** Blue channel C(cp/µL)	Nb positive droplets	**B** Green channel C(cp/µL)	Nb positive droplets	**C** % unbroken CTG	**D** % broken CTG
Double population (unbroken CTG + *JEM1*)	20 227	668	6891	–	–	Column (A_unbroken CTG_/B_*JEM1*_) x 100 = 93.7%	100-Column C = 6.3%
Single population (*JEM1*)	20 227	348.4	3950	344.9	3 914		
Single population (unbroken CTG)	20 227	323.1	2977	–	–		

a Samples #1 and #2 correspond to blots quantified in Fig. 2C and D and Table 1.

Note that the fluorescence detection threshold in the blue channel can be adjusted to consider only the upper population representing *JEM1* amplification. The number of DNA molecules of *JEM1* was found to be the same in the blue channel and in the green channel (Table 2). This also serves as a control, and we can see that a single-copy gene amplification through a probe or with EvaGreen leads to the same results. Quantifications obtained by Southern blot and dPCR for the same sample with the same percentage of DSB were compared and shown to be very similar: 31.2% DSB by Southern blot and 34.6% DSB by dPCR for DNA sample #1, 7.8% by Southern blot and 6.3% by dPCR for DNA sample #2 (Tables 1 and 2). This validated our dPCR protocol.

## Discussion

In the present work, we have compared four different molecular methods to quantify DSBs in budding yeast genomic DNA. We found that dPCR was at the same time precise and less time-consuming than alternative methods. It even allows to detect very small percentage of breaks. It is easy to set up and the experiment can be performed within a day (Table 3). In addition, the results found by dPCR are identical to results found by Southern blot. Our protocol potentially has applications for DSB quantification within repetitive elements, non-B DNA, fragile sites, as well as in non-repetitive DNA.

**Table 3.** Comparison of each method.

Method	Southern blot	DSB-PCR	DSB-qPCR	dPCR
Time	Long (3–4 days)	Short (1 day)	Short (1 day)	Short (1 day)
Cost	85€/blot	3€/reaction	4.5€/reaction	8.3€/reaction
Recombination intermediates	Visible	Not visible	Not visible	Not visible
DSB quantification	Yes	No	Yes	Yes

Among former methods used to quantify telomere length, Monochrome Multiplex quantitative PCR (MMqPCR) used specific sets of primers to measure human telomere length. Two amplicons in the same reaction are detected, one is a telomeric sequence (T) and the other is a single-copy gene (S), albumin, used as a control. The ratio of T/S is proportional to the average telomere length [27]. It was shown that the MMqPCR was more in line with results obtained by Southern blot than with singleplex PCR [27]. O’Callaghan and Fenech described a modification of the MMqPCR method [28], in which a standard oligomer of a single-copy gene is added to the reaction at a defined concentration and amplified using dedicated primers. The use of a standard oligomer shared by all laboratories allows to get a more direct comparison of results between experiments and among labs. Another approach was to ligate a specific oligomer to 3ʹ-OH telomeric extensions, followed by amplification with a complementary primer and a subtelomere-specific primer. This was combined with PacBio sequencing to study sequence variation among *Schizosaccharomyces pombe* telomeric repeats [36]. PacBio has been recently shown to be an efficient technology to sequence long repeats [23, 37]. In our case, the use of DSB-PCR is not quantitative enough to determine the proportion of broken DNA molecules. Therefore, we switched to DSB-qPCR, which needs the determination of a standard curve to quantify the DSB. The correlation coefficient was not optimal (0.869), probably due to the low DNA concentration used (0.15 ng/µL per qPCR reaction), which is 7 times less than the modified MMqPCR method [28]. To improve quantification reliability, we therefore recommend a minimum DNA concentration of 1 ng/µL per qPCR reaction. Nonetheless, DSB-qPCR quantification was not as precise as dPCR. Moreover, a new standard curve must be generated for every new application at a new genomic location with new primers.

In our dPCR experiments, the uncut CTG repeats were amplified by EvaGreen. The control gene was also amplified with the same dye and quantified using a specific HEX probe. A similar approach could not be used on CTG triplet repeats, since such a probe would form secondary structures that would impede correct hybridization to its target, in addition to possible multiple bindings at several positions on the repeat tract, biasing detection and quantification. Note that the HEX probe is not essential, since two populations are seen in the blue channel, representing the *JEM1* gene amplification as well as the uncut CTG amplification. By modifying the fluorescence detection threshold in this channel, one can determine the exact number of molecules in each population without the need for the HEX probe. Therefore, while the HEX probe is not essential for quantification and could be removed for further applications, it can still be included as an additional control to verify *JEM1* detection and to check the reproducibility between EvaGreen and the HEX probe.

### Strengths and limitations of our method

In summary, we have compared four methods (Southern blot, DSB-PCR, DSB-qPCR, and dPCR) to quantify a single DSB in a long repeated and structured sequence. We have developed a new protocol to precisely quantify a DSB in this DNA context by dPCR. This method addresses a common concern with PCR-based assays targeting structured or repetitive DNA regions, where signal loss may reflect poor amplification rather than true breaks. In our case, control experiments using qPCR and dPCR with dilutions of DNA with known DSB content confirmed high amplification efficiency of the CTG-spanning primers (Supplemental Figs. S1A-B), and the strong concordance with Southern blot measurements (Tables 1 and 2) supports that signal loss in our dPCR assay reliably reflects DSBs. This method is therefore potentially useful to study fragile sites or long repeated sequences encompassing DNA secondary structures, which may impede enzymatic reactions used in other methods. Although the present work was performed in yeast with TALEN-induced breaks, the method is not restricted to this system. It could be adapted to human cells, with optimization of the DNA input per dPCR reaction to account for genome size and complexity. Moreover, TALENs are not the only nucleases suitable for DSB induction. CRISPR-Cas9, which is easier to design and widely adopted in mammalian systems, can also generate targeted breaks that could be fully compatible with this assay. dPCR could thus be broadly applied to study DSBs induced by various systems, including CRISPR-Cas9, across diverse genomic contexts and in different cell types, such as human cells.

Our protocol does not allow to assess the presence of multiple DSBs at different locations within the target sequence. New primers used to amplify a specific repeated sequence need to be checked before the experiment to determine if they can be used to amplify the repeated sequence quantitatively and efficiently. Note that there is a limit to the length that may be amplified by dPCR. In our study, 80 CTG triplets did not impede the dPCR amplification; however, much longer repeats might prove harder to be amplified.

## Supplementary Material

bpaf059_Supplementary_Data

## Data Availability

Raw data are available here (Zenodo DOI: 10.5281/zenodo.14507710).
