# Supplementary material for: Fast and accurate quantification of double-strand breaks in microsatellites by digital PCR
Source: Biol Methods Protoc. 2025 Aug 9;10(1):bpaf059. doi: 10.1093/biomethods/bpaf059 (PMC12377901; doi:10.1093/biomethods/bpaf059)
Supplement: bpaf059_Supplementary_Data [file bpaf059_supplementary_data.docx]

**Supplementary data**

**Supplemental Table S1**.

| **Oligonucleotide** | **Sequence** |
| --- | --- |
| su37 | 5’- TTG AGT CCA ATG CAC ATA GT -3’ |
| su38 | 5’- CTT ACG TCG TTT TGC AGT TT -3’ |
| su39 | 5’- TCC TTC CAA GAT GGT TCA GA -3’ |
| su40 | 5’- TAT GGA AAA TAA CGC AGC AG -3’ |
| G18 | 5’- CGG GAT CCG18 -3’ |
| VMS14 | 5’- GAC GTA AGA CAA GTA CAG TAG AAT GAA TGG CTG CGC TTC ACC ACC AAT G -3’ |
| CP19 | 5’- AAG AAA TGG TCT GTG ATC CCC C -3’ |
| upCTGfwd | 5’- CTC GGT AGC CAA GTT GGT TTA AG -3’ |
| CP21 JEM1f | 5’- TGT GAT TTG GCT GAG TTA CAA CG -3’ |
| CP22 BIS JEM1 | 5’- ATG CAC CTT GTC CCA CAT CC -3’ |
| JEM1 probe | 5’- [HEX]G GAT CGC TGG GCA GTT GGA ACA AAA CTG CAT[MGB-Eclipse Quencher] -3’ |


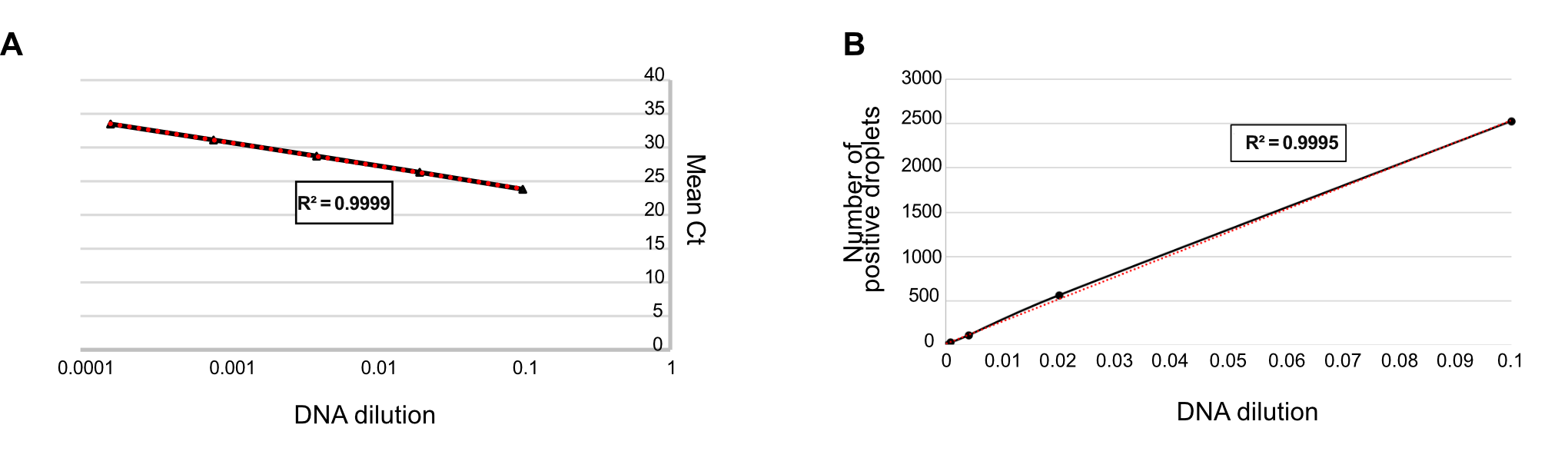


**Supplemental Figure S1.**
